# Evolution of journal rankings in orthopedics and sports medicine (2000-2024): A SCImago-based bibliometric analysis

**DOI:** 10.1007/s00132-025-04683-y

**Published:** 2025-07-31

**Authors:** Raju Vaishya, Sudhir Shekhawat, Abhishek Vaish, Filippo Migliorini

**Affiliations:** 1 Department of Orthopaedics and Joint Replacement Surgery, Indraprastha Apollo Hospitals, 110076 New Delhi, India; 2 Department of Trauma and Reconstructive Surgery, University Hospital of Halle, Martin-Luther University Halle-Wittenberg, 06097 Halle (Saale), Germany; 3 Department of Orthopaedic and Trauma Surgery, Academic Hospital of Bolzano (SABES-ASDAA), 39100 Bolzano, Italy; 4 Department of Life Sciences, Health, and Health Professions, Link Campus University, 00165 Rome, Italy

**Keywords:** Bibliometrics, Impact factor, Citation analysis, Academic publishing, Research trends, Bibliometrische Daten, Impaktfaktor, Zitieranalyse, Akademisches Publizieren, Forschungstrends

## Abstract

**Background:**

Academic journals are fundamental to scientific communication, facilitating the dissemination of research findings that spur innovation across clinical and scientific fields. Journal rankings provide valuable insights into the influence and credibility of scholarly outlets.

**Objective:**

This study analyzes the evolution of journal rankings in orthopedics and sports medicine (OSM) from 2000 to 2024, using SCImago Journal Rank (SJR) data to uncover trends in journal prestige and regional representation.

**Material and methods:**

A comprehensive bibliometric analysis was performed using SCImago data, comparing journal rankings over 25 years. Key metrics included SJR values and citation counts across orthopedic and sports medicine journals. Statistical significance was assessed using an Independent samples Kruskal-Wallis test.

**Results:**

The analysis revealed a significant average increase of 36.6% in the SJR of leading OSM journals over the study period. Notably, only 12% of the top ranking journals originated from non-western regions, indicating a geographic bias. Sports medicine-focused journals showed a higher average SJR of 1.75, compared to 1.40 for orthopedic journals (*p* < 0.05). A global median of 28.7% of female authors was found, reflecting ongoing gender disparities in authorship within OSM.

**Conclusion:**

The findings highlight marked shifts in journal prestige in the OSM field over 25 years, alongside persistent regional biases that may disadvantage high-quality research from non-western regions. The study underlines the importance of awareness regarding these dynamics for stakeholders in making decisions related to publication strategies and funding. Future research should further explore the impact of emerging subfields and the role of open-access publishing in shaping journal rankings in OSM.

**Graphic abstract:**

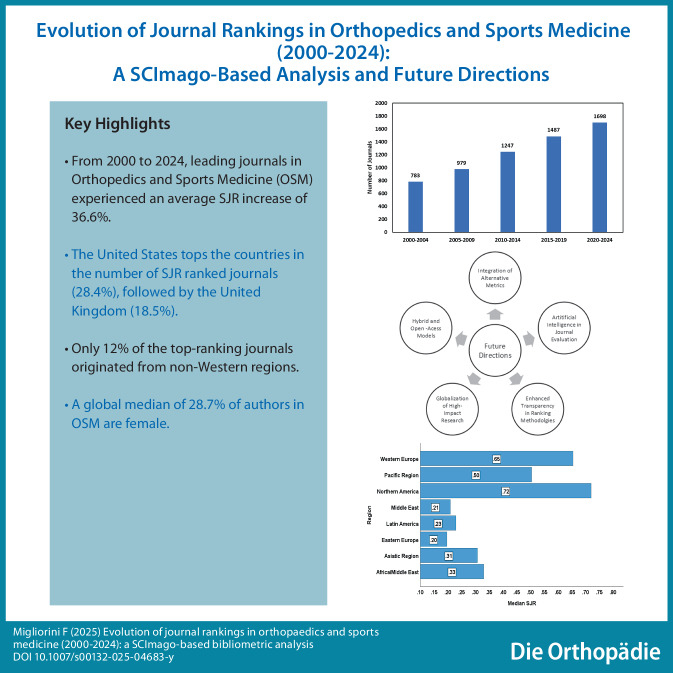

**Supplementary Information:**

The online version of this article (10.1007/s00132-025-04683-y) contains supplementary material, which is available to authorized users.

## Abstract (de)

**Hintergrund:**

Akademische Fachzeitschriften sind von grundlegender Bedeutung für die wissenschaftliche Kommunikation, da sie die Verbreitung von Forschungsergebnissen erleichtern und die Innovationen in allen klinischen sowie wissenschaftlichen Bereichen vorantreiben. Zeitschriftenrankings bieten wertvolle Einblicke in den Einfluss und die Glaubwürdigkeit wissenschaftlicher Publikationen.

**Zielsetzung:**

In dieser Studie wird die Entwicklung der Zeitschriftenrankings im Bereich Orthopädie und Sportmedizin (OSM) von 2000 bis 2024 analysiert. Dabei werden die Daten des SCImago Journal Rank (SJR) verwendet, um Trends in Bezug auf das Prestige und die regionale Vertretung von Zeitschriften aufzuzeigen.

**Material und Methoden:**

Es wurde eine umfassende bibliometrische Analyse unter Verwendung von SCImago-Daten durchgeführt, bei der die Ranglisten der Zeitschriften über 25 Jahre hinweg verglichen wurden. Zu den wichtigsten Metriken gehörten die SJR-Werte und die Anzahl der Zitate in orthopädischen und sportmedizinischen Fachzeitschriften. Die statistische Signifikanz wurde mit einem Kruskal-Wallis-Test für unabhängige Stichproben ermittelt.

**Ergebnisse:**

Die Analyse ergab einen signifikanten durchschnittlichen Anstieg des SJR der führenden OSM-Zeitschriften um 36,6 % im Untersuchungszeitraum. Bemerkenswert ist, dass nur 12 % der führenden Zeitschriften aus nichtwestlichen Regionen stammten, was auf eine geografische Verzerrung hinweist. Sportmedizinisch ausgerichtete Zeitschriften wiesen eine höhere durchschnittliche SJR von 1,75 auf, verglichen mit 1,40 für orthopädische Zeitschriften (*p* < 0,05). Der Medianwert der weiblichen Autoren lag bei 28,7 %, was auf die anhaltenden geschlechtsspezifischen Ungleichheiten bei der Autorenschaft in der OSM hinweist.

**Schlussfolgerung:**

Die Ergebnisse verdeutlichen, dass sich das Ansehen von Fachzeitschriften im OSM-Bereich in den letzten 25 Jahren deutlich verändert hat und dass es nach wie vor regionale Vorurteile gibt, die qualitativ hochwertige Forschung aus nichtwestlichen Regionen benachteiligen können. Die Studie unterstreicht, wie wichtig es für die Beteiligten ist, sich dieser Dynamik bewusst zu sein, wenn sie Entscheidungen in Bezug auf Publikationsstrategien und Finanzierung treffen. Künftige Forschungsarbeiten sollten die Auswirkungen neu entstehender Teilbereiche und die Rolle des Open-Access-Publizierens bei der Gestaltung von Zeitschriftenrankings im OSM-Bereich weiter untersuchen.

## Introduction

Academic journals are fundamental to scientific communication, facilitating the dissemination of research findings that spur innovation across clinical and scientific fields [1, 2]. In orthopedics and sports medicine (OSM), where advancements in surgical techniques, rehabilitation protocols and biomechanical research evolve rapidly, choosing suitable journals for publication is vital for researchers, clinicians and institutions [3]. Journal rankings, typically derived from bibliometric indicators, provide valuable insights into the influence and credibility of scholarly outlets, which in turn guide stakeholders in making decisions regarding publication strategies, funding allocations and academic promotions [4].

The SCImago Journal Rank (SJR) has emerged as a significant metric for evaluating journal quality, using Scopus citation data to assess a journal’s prestige based on weighted citations [5]. In contrast to the Journal Impact Factor (JIF), which merely counts citations per article, the SJR considers the significance of the citing journals, providing a more nuanced evaluation of influence [6]. While prior studies have focused on journal rankings in various medical specialities, few have conducted a longitudinal analysis of OSM journals using SCImago data over an extended period (2000–2024). Furthermore, existing literature often overlooks the evolving landscape of open-access (OA) publishing, the geographic disparities in journal representation and the comparative performance of journals focused on general orthopedics versus sports medicine [7].

Despite widespread use of bibliometric indicators, research gaps remain. Specifically, there is limited understanding of how OSM journal rankings have changed, especially concerning emerging subfields, such as regenerative medicine. Additionally, the impact of OA publishing on journal rankings is underexplored [8] as are the effects of geographical biases that could disadvantage high-quality research from non-western regions [9].

This study aims to fill these gaps by providing a comprehensive SCImago-based evaluation of OSM journal rankings from 2000 to 2024. Thus, it aids informed decision-making for researchers and policymakers and enriches discussions around bibliometric indicators as measures of journal quality.

## Material and methods

### Data collection

The SCImago website [10] was searched on 1 May 2025 and the data related to the latest global journal rankings and values in OSM were collected using the following strategy: *SCImago>> Journal Rankings>>All Subject Areas>>Orthopedics and Sports Medicine>>All Regions>>Types (Journals)>>Year (2000–2024). *The data were downloaded as CSV files and then exported to the SPSS data editor (SPSS Software version 25 (IBM SPSS Inc, Chicago, USA)), from which the data analysis was done. The dataset comprises 335 journals categorized under OSM. Bibliometric data were extracted and analyzed, including SJR, H‑index and publication and citation counts over 3 years (Supplementary Table 1). Additionally, information on the gender distribution of authors, the geographic location of the journals and their respective publishers was incorporated into the analysis. To assess the relative impact and productivity of the journals, they were stratified into quartiles (Q1, Q2, Q3, and Q4) based on the SJR scores. The SJR quartiles measure a journal’s influence and prestige, with Q1 being the highest (most influential) and Q4 being the lowest.

### Statistical analysis

Descriptive statistics were used to summarize the data. Tests of normality of quantitative data were assessed by applying the Kolmogorov-Smirnov test. Given the non-normal data, results are presented as medians and interquartile ranges. Independent samples Kruskal-Wallis tests were used for group comparisons. The interquartile range (IQR) was defined by the values at the first quartile (Q1) and the third quartile (Q3). The correlation analyses were performed to assess the relationship of SJR with other bibliometric indicators. A *p*-value < 0.05 was considered significant. All analyses were performed using IBM SPSS Statistics version 29.0, Armonk, NY, USA.

## Results

### Growth of SJR ranked journals (2000–2024)

**Fig. 1:**
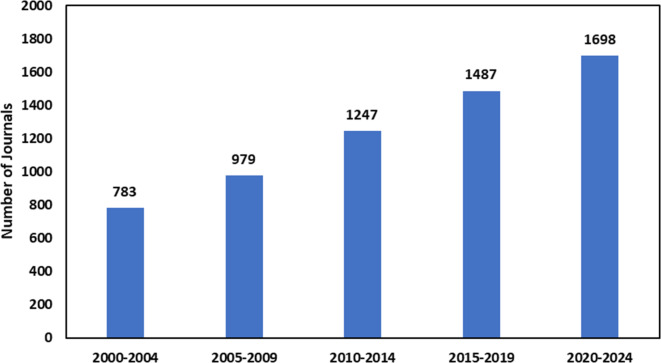
Trend of publications of journals (2000–2024)—Source: SCImago

The total number of journals with an SJR ranking between 2000 and 2024 reached 6194, starting from 140 journals in the year 2000 and growing to 335 by 2024 (Fig. 1), with a 58.1% increase. An average increase of 36.6% in the SJR of the OSM journals was observed over the study period.

### Regional distribution of the journals

The analysis of SJR-ranked journals reveals notable regional disparities, with Western Europe leading in the top Q1 quartile with 44 journals and North America with 35, indicating a strong concentration of high-impact publications in these areas. While other regions have fewer journals in Q1, Western Europe boasts the highest number of journals at 143, and North America follows with a substantial 95 (Table 1). Specific regional insights show that the Africa/Middle East and Pacific regions have few top-quartile journals and low total numbers. In contrast, Asia has a more even distribution but fewer Q1 journals than Western and Northern America. Latin America and the Middle East show a trend of increasing journal numbers in the lower quartiles.

**Table 1:** Distribution of journals across different regions and their classification into SJR (SCImago Journal Rank) quartiles in 2024

Region	SJR Quartile				Total
	Q1	Q2	Q3	Q4	
Africa/Middle East	0	0	2	1	3
Asiatic Region	2	12	6	15	35
Eastern Europe	1	0	11	21	33
Latin America	1	1	2	5	9
Middle East	1	2	2	10	15
North America	35	27	26	7	95
Pacific Region	0	1	1	0	2
Western Europe	44	41	33	25	143
*Total*	*84*	*84*	*83*	*84*	*335*

**Fig. 2:**
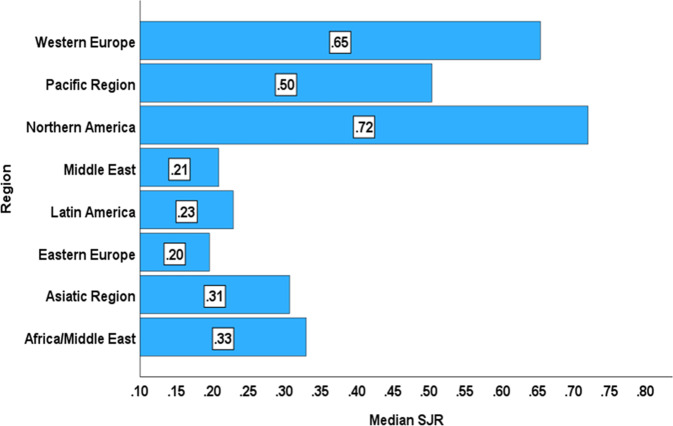
SCImago Journal Ranking (SJR) quartile and median ranking across global journal regions

While Western Europe hosts the largest number of journals (*n* = 143, 42.7%), followed by North America (*n* = 95, 28.4%), North America exhibits the highest median SJR ranking (0.72) compared to Western Europe (0.65). Notably, the Pacific Region has no Q1 journals but maintains a median SJR ranking of 0.50. Emerging regions, including Asia (*n* = 35, 10.4%), Eastern Europe (*n* = 33, 9.9%), the Middle East (*n* = 15, 4.5%) and Latin America (*n* = 9, 2.7%), contribute a smaller proportion of the total journals, while Africa/Middle East has the fewest journals (*n* = 3, 0.9%) and a median SJR ranking of 0.33 (Fig. 2).

### Top countries in SJR ranked journals

The top 10 countries in the number of journals with SJR ranking are dominated by the USA (US), which stands out with 95 journals, representing 28.4% of this group. Western Europe also has a strong presence, with the United Kingdom (UK) being the second highest at 62 journals (18.5%), alongside Germany, the Netherlands, Italy and France. South Korea (10 journals) and China (11 journals) from the asiatic region are represented, indicating their growing contribution to global academic publishing; however, their numbers are lower than those of the USA and UK. Turkey and the Russian Federation represent the Middle East and Eastern Europe, respectively, with fewer journals in the top 10. Notably, the US and UK together account for nearly half (46.9%) of the journals from these leading nations. Regarding influence, the UK leads with the highest median SJR of 0.846, suggesting a higher average prestige of its journals, while the USA also has a relatively high median SJR of 0.720. In contrast, countries like China, Turkey, and the Russian Federation exhibit considerably lower median SJR scores. Within Western Europe, Germany and the Netherlands have moderate median SJR values, while Italy and France have slightly lower ones (Table 2).

**Table 2:** Top 10 countries in number of journals with the highest SCImago Journal Ranking (SJR) in 2024

Country	Region	Number	Percentage (%)	Median SJR
United States	North America	95	28.4	0.720
United Kingdom	Western Europe	62	18.5	0.846
Germany	Western Europe	25	7.5	0.538
Netherlands	Western Europe	17	5.1	0.567
Italy	Western Europe	11	3.3	0.450
France	Western Europe	10	3.0	0.410
South Korea	Asiatic Region	10	3.0	0.475
China	Asiatic Region	11	3.3	0.167
Turkey	Middle East	8	2.4	0.197
Russian Federation	Eastern Europe	10	3.0	0.145

### Bibliometric indicators and SJR quartile correlation

A statistically significant trend (*p* < 0.001) across various bibliometric indicators (Table 3) highlights the effectiveness of SJR quartiles in differentiating journals based on impact and productivity. The Q1 journals boast the highest median H‑index at 74.5, decreasing to 47.5 in Q2, 25.0 in Q3, and 9.0 in Q4. This hierarchy is reflected in publication figures, with Q1 journals having a median of 178 total documents in 2024 and 442 over 3 years, significantly outperforming the lower quartiles. The Q1 journals also have a higher median for total references (*n* = 6860) and total cites over 3 years (*n* = 1564) than Q4’s median of 46 citations.

**Table 3:** Key bibliometric indicators by SCImago Journal Ranking (SJR) quartiles

Parameters		Total	Q1	Q2	Q3	Q4	*p*-value
H‑index	Median	33.0	74.5	47.5	25.0	9.0	< 0.001
	IQR	(13.0–62.0)	(50.0–148.8)	(28.0–72.3)	(14.0–40.0)	(5.8–9.0)	
Total documents (2024)	Median	72	178	91	57	43	< 0.001
	IQR	(39–154)	(92–305)	(49–172)	(31–94)	(25–69)	
Total documents (3 years)	Median	208	442	266	155	127	< 0.001
	IQR	(113–428)	(241–808)	(154–471)	(96–278)	(90–197)	
Total references	Median	2537	6860	3494	1722	1152	< 0.001
	IQR	(1270–5252)	(3918–11436)	(1712–5817)	(995–2802)	(718–1882)	
Total cites (3 years)	Median	305	1564	546	172	46	< 0.001
	IQR	(88–1055)	(907–2998)	(319–1070)	(106–273)	(21–84)	
Citable documents (3 years)	Median	196	414	243	140	118	< 0.001
	IQR	(105–397)	(213–693)	(144–456)	(89–264)	(85–174)	
Cites per document (2 years)	Median	1.6	3.3	1.9	1.1	0.3	< 0.001
	IQR	(0.6–2.6)	(2.3–4.3)	(1.6–2.3)	(0.7–1.5)	(0.2–0.5)	
Reference per document	Median	34.6	38.6	34.5	36.0	29.1	< 0.001
	IQR	(27.7–41.5)	(32.1–49.5)	(30.4–44.1)	(25.4–40.9)	(22.1–36.5)	

Table 4 illustrates a clear hierarchical trend in journal impact and productivity across quartiles, with Q1 journals exhibiting the highest average SJR (1.45), H‑index (103.9), number of publications (591) and citations (2250) over the past 3 years, followed by a consistent decline in these metrics from Q2 to Q4. Notably, while the number of journals is relatively consistent across all quartiles (83–84), the average female authorship shows a slight variation, with Q3 journals having the highest average (32.6%) and Q2 the lowest (27.9%), suggesting that journal quartile is strongly associated with impact and output but has a less direct relationship with the proportion of female authors.

**Table 4:** Average Bibliometric indicators by journal quartile

Quartile	Number of journals	Average SJR	Average H‑index	Average publications (3 years)	Average citations (3 years)	Average female authorship (in %)
*Q1*	84	1.45	103.9	591	2250	30.4
*Q2*	84	0.70	55.7	404	913	27.9
*Q3*	83	0.36	31.0	217	270	32.6
*Q4*	84	0.16	12.3	212	74	31.5

**Fig. 3:**
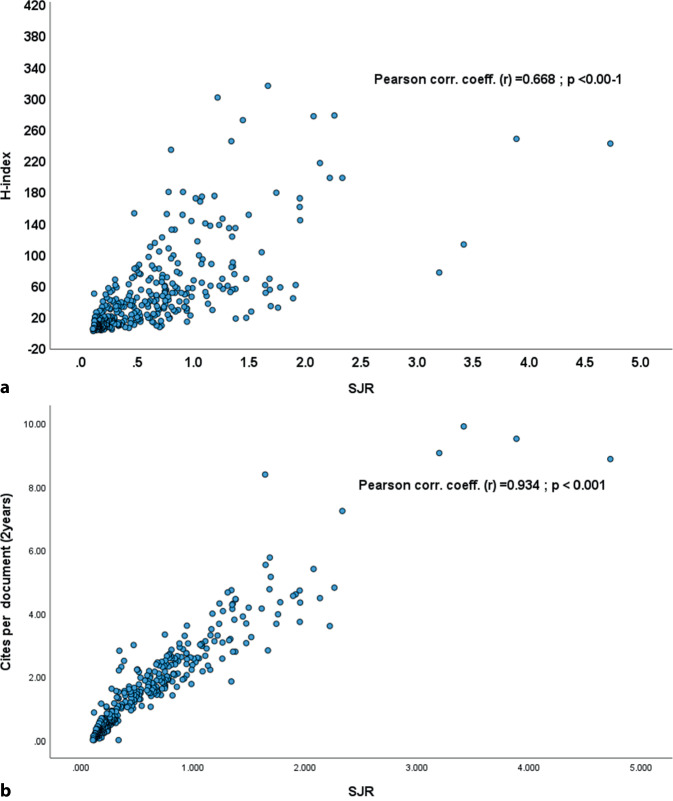
**a** Scatter plot for SJR and H‑index showing high correlation, **b** scatter plot for SJR and cites per document (2 years) showing high correlation

Correlation analysis shows high correlations between SJR and H‑Index (correlation coefficient = 0.668; *p* < 0.001) and between SJR and CPD (correlation coefficient = 0.934; *p* < 0.001) (Fig. 3a, b).

### Regional distribution of female authors

The female authorship across regions revealed significant variability within most areas, as demonstrated by the wide ranges between minimum and maximum percentages; for instance, Eastern Europe spans from 0.0% to 75.0% (Table 5). Despite this internal variation, the median rate of female authors across all regions is relatively consistent, hovering between the high 20%s and low 30%s, with a global median of 28.7%, indicating a persistent underrepresentation. Regions with smaller IQRs, such as Africa/Middle East and the Pacific Region (based on only 3 and 2 journals), suggest more homogeneity in female authorship within their journals; however, the small sample sizes limit generalizability.

**Table 5:** Regional distribution of female authors

Region	Number of journals	Minimum (%)	Maximum (%)	Median (%)	Interquartile range (IQR; %)	
					Q1	Q3
Africa/Middle East	3	26.1	40.0	26.5	26.1	–
Asiatic Region	35	0.0	55.1	23.3	16.5	31.2
Eastern Europe	33	0.0	75.0	34.7	22.6	45.4
Latin America	9	17.7	62.1	28.7	19.9	60.4
Middle East	15	11.1	59.6	32.3	20.1	42.1
Northern America	95	8.9	68.4	28.6	20.9	41.4
Pacific Region	2	25.4	34.7	30.0	25.4	–
Western Europe	143	0.0	65.3	28.9	22.8	37.8
Total	335	0.0	75.0	28.7	21.5	38.1

Conversely, regions with larger IQRs, including the Asiatic Region, Eastern Europe, North America, and Western Europe, show greater diversity in female author representation. Examining regional medians, Eastern Europe exhibits the highest at 34.7%, while the Asian Region has the lowest at 23.3%. Other regions like Latin America, the Middle East, North America, and Western Europe have median percentages closer to the global average. The Q1 and Q3 values further clarify the distribution; for example, in the Asiatic Region, 25% of journals have 16.5% or fewer female authors, and 75% have 31.2% or fewer, contrasting with Eastern Europe where 25% have 22.6% or fewer, and 75% have 45.4% or fewer. The findings for Africa/Middle East and the Pacific Region should be interpreted cautiously given the limited number of journals analyzed.

### Top 10 most influential journals in OSM

The top 10 most influential journals (Table 6), as ranked by SJR, reveal the *British Journal of Sports Medicine* as the leader with the highest SJR (4.724), although *Sports Medicine* boasts a slightly higher H‑index (247). Generally, higher SJR values tend to align with higher H‑indices. Yet, exceptions like the *American Journal of Sports Medicine *and the *Journal of Bone and Mineral Research*, which have very high H‑indices but lower SJR values than others, suggest different patterns of citation impact. The subject matter is heavily skewed towards sports medicine and related fields, with four journals explicitly mentioning it, alongside a strong presence of journals focused on bone and joint health. The SJR values range from 4.724 to 1.954, and H‑indices vary significantly from 76 to 277, illustrating a spectrum of influence and long-term impact among these leading publications in their respective domains.

**Table 6:** Top 10 most influential journals based on SCImago Journal Ranking (SJR)

Ranking	Journal	SJR	H‑Index
1	*British Journal of Sports Medicine*	4.724	241
2	*Sports Medicine*	3.887	247
3	*Journal of Cachexia, Sarcopenia and Muscle*	3.416	112
4	*Journal of Sport and Health Science*	3.197	76
5	*Osteoarthritis and Cartilage*	2.331	197
6	*American Journal of Sports Medicine*	2.260	277
7	*Arthroscopy—Journal of Arthroscopic and Related Surgery*	2.219	197
8	*Bone and Joint Journal*	2.131	216
9	*Journal of Bone and Mineral Research*	2.074	276
10	*Spine Journal*	1.954	143

### Impact, concentration and publisher influence

The analysis indicates a concentration of high-impact journals (Q1) in North America and Western Europe, with the UK and the US leading in the number of Q1 journals. This suggests a strong influence of research institutions and funding structures in these regions on the global landscape of OSM research.

### Representation of emerging regions

Asia, Latin America, and Africa have less representation of journals in the top quartiles. This observation underscores potential disparities in research funding, infrastructure, and established academic networks that may affect the visibility and impact of research originating from these regions; however, this also highlights significant opportunities for growth and increased representation in the global scholarly landscape.

## Discussion

The evolution of journal rankings in OSM from 2000 to 2024, as evidenced by this SCImago-based analysis, reveals significant trends that shape the landscape of academic publishing in this field. The findings indicate an average increase of 36.6% in the SJR of leading OSM journals over the past 25 years, demonstrating a sustained enhancement in prestige and impact among these scholarly publications [11–13].

Analysis of the geographical representation within OSM journal rankings reveals a concerning disparity, with only 12% of the top ranking journals originating from non-Western regions. This stark underrepresentation of international perspectives in leading journals is a critical issue for the field. The limited inclusion of non-Western journals may overshadow valuable research contributions that could provide diverse insights and innovations in OSM globally. Addressing this bias necessitates strategic efforts from publishers and researchers to foster inclusivity and ensure that a broader array of voices is represented in prominent journals. Enhancing the representation of non-Western research enriches academic discourse and aligns with the global nature of healthcare practices and challenges [14–16].

Comparative analysis between OSM-focused journals further elucidates the dynamics within the field, revealing that sports medicine journals achieved an average SJR of 1.75 compared to 1.40 for orthopedic journals, with this distinction proven statistically significant (*p* < 0.05). This difference sheds light on potential variations in research output, funding, and academic interest across these subdisciplines, suggesting that while both areas contribute significant advancements to healthcare, sports medicine is currently more successful in garnering recognition [17–19]. This opens avenues for further investigation into why certain research areas attract higher prestige and how factors such as funding sources, institutional support, and the perceived impact of research can be optimized to elevate orthopedic journals [20].

The use of OA publishing had a pronounced impact on journal prestige and there were higher citations than in traditional journals. This shift signifies changing dynamics in academic dissemination, emphasizing the growing relevance of OA models in the digital age. The rise of OA publishing indicates a more inclusive approach to scientific communication, enabling wider access to research findings and facilitating rapid knowledge exchange. As OA models evolve, they present opportunities for researchers to reach broader audiences, thereby enhancing their disciplines’ visibility [21–23].

The SJR is a valuable metric that assesses a journal’s scientific influence, considering the number of citations received and the prestige of the citing journals [24–26]. Unlike the Journal Impact Factor (JIF), which counts all citations equally over a 2-year window of articles and reviews, SJR uses a 3-year citation window and considers all document types indexed in Scopus. Furthermore, SJR normalizes citation counts across different subject fields, enabling more equitable comparisons between disciplines, a feature not present in the JIF. While JIF relies on Web of Science data, SJR is based on the broader Scopus database and is freely accessible, offering a wider coverage of journals, including those from diverse countries and languages [5, 27].

While the findings offer valuable insights, it is crucial to acknowledge certain limitations inherent in this study. Our focus on SCImago data means that the analysis did not include other bibliometric indicators, such as alternative citation metrics, qualitative assessments of journal quality, and field-specific citation contexts. This limitation may influence our understanding of comprehensive journal quality and impact. Future research should aim to expand the scope of inquiry to incorporate various bibliometric indicators, including altmetrics, and undertake qualitative analyses to provide a more nuanced view of journal rankings [28].

**Fig. 4:**
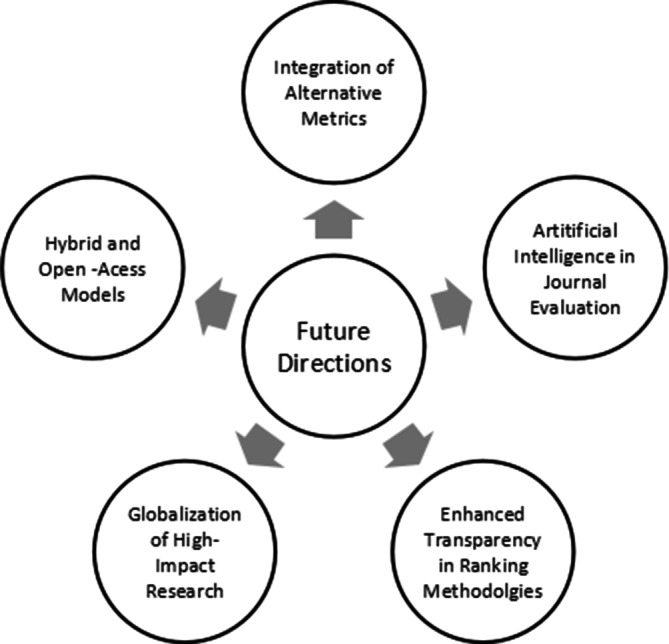
Future directions in journal rankings

Additionally, this study prompts consideration of future directions in both research and publishing practices (Fig. 4). As the influence of OA publishing continues to grow, it will be essential to monitor how this impacts traditional journal models and the OSM field as a whole. Researchers may find that funding allocations favor OA models, incentivising high-quality research to be published in more accessible formats. Furthermore, as emerging fields and methodologies transform OSM, scholars should remain adaptable, ready to actively engage with these changes and to explore new platforms that could enhance their work’s impact.

The findings of this study encourage ongoing dialogue about the dynamics of journal rankings and the implications for the academic publishing landscape in OSM. By understanding these shifting trends, researchers, institutions, and policymakers can collaborate to advance the visibility and impact of high-quality research initiatives, fostering a more inclusive and dynamic academic environment.

## Conclusion

This study reveals significant advancements in the prestige of leading journals in orthopedics and sports medicine (OSM) over the past 25 years, with an average SJR increase of 36.6%; however, it also highlights a notable geographic disparity, as only 12% of top ranking journals come from non-Western regions, indicating a potential bias in representation. Sports medicine journals generally outperform orthopedic journals regarding SJR, averaging 1.75 compared to 1.40. Additionally, female authors make up a global median of 28.7% of contributors in OSM, reflecting ongoing gender disparities. These findings highlight the need for stakeholders to consider these trends when forming publication strategies and funding decisions, and the importance of addressing inequalities to ensure a more inclusive academic landscape.

## Supplementary Information

Supplementary Table 1

## Data Availability

The datasets generated during and/or analyzed during the current study are available throughout the manuscript.

## Declarations

For this article no studies with human participants or animals were performed by any of the authors. All studies mentioned were in accordance with the ethical standards indicated in each case.
