# Supplementary material for: Evolution of journal rankings in orthopedics and sports medicine (2000-2024): A SCImago-based bibliometric analysis
Source: Orthopadie (Heidelb). 2025 Jul 31;54(10):795–803. doi: 10.1007/s00132-025-04683-y (PMC12457506; doi:10.1007/s00132-025-04683-y)
Supplement: Supplementary file 1 — Supplementary Table 1 [file 132_2025_4683_MOESM1_ESM.pdf]

Rank	Title	SJR	SJR Quartile	Hindex	Total Docs 2024	Total Docs 3years	Total Refs	Total Cites 3years	Citable Docs 3years	Cites Doc 2years	Ref. Doc	% Female	Country	Region
1	British Journal of Sports Medicine	4,724	Q1	241	289	877	9811	7484	530	8,86	33,95	44,73	United Kingdom	Western Europe
2	Sports Medicine	3,887	Q1	247	214	520	18981	5413	432	9,50	88,70	29,08	Switzerland	Western Europe
3	Journal of Cachexia, Sarcopenia and Muscle	3,416	Q1	112	254	694	10047	7129	633	9,89	39,56	37,94	United States	Northern America
4	Journal of Sport and Health Science	3,197	Q1	76	109	244	5485	2359	207	9,05	50,32	37,93	Netherlands	Western Europe
5	Osteoarthritis and Cartilage	2,331	Q1	197	185	533	8681	3789	456	7,23	46,92	42,45	United Kingdom	Western Europe
6	American Journal of Sports Medicine	2,260	Q1	277	412	1361	15678	6904	1202	4,81	38,05	22,61	United States	Northern America
7	Arthroscopy - Journal of Arthroscopic and Related Surgery	2,219	Q1	197	690	1450	19342	4890	796	3,60	28,03	19,84	United States	Northern America
8	Bone and Joint Journal	2,131	Q1	216	251	687	9066	3388	633	4,48	36,12	20,02	United Kingdom	Western Europe
9	Journal of Bone and Mineral Research	2,074	Q1	276	186	653	7291	3500	571	5,40	39,20	43,36	United States	Northern America
10	Spine Journal	1,954	Q1	143	308	717	10584	3680	625	4,34	34,36	21,58	United States	Northern America
11	Journal of Arthroplasty	1,951	Q1	171	851	1933	27126	7466	1791	3,73	31,88	20,81	United States	Northern America
12	Knee Surgery, Sports Traumatology, Arthroscopy	1,950	Q1	160	438	1638	16545	7594	1562	4,72	37,77	22,28	Germany	Western Europe
13	Skeletal Muscle	1,914	Q1	60	37	75	2174	432	75	4,60	58,76	50,00	United Kingdom	Western Europe
14	Knee Surgery and Related Research	1,891	Q1	43	47	119	1663	529	118	4,55	35,38	21,12	United Kingdom	Western Europe
15	EFORT Open Reviews	1,776	Q1	57	109	292	6663	1568	289	4,35	61,13	23,77	United Kingdom	Western Europe
16	JBJS Open Access	1,756	Q1	31	95	240	3185	1066	234	3,97	33,53	25,19	United States	Northern America
17	Journal of Shoulder and Elbow Surgery	1,741	Q1	178	488	1245	17036	5087	1194	3,67	34,91	21,76	United States	Northern America
18	Science and Medicine in Football	1,691	Q1	33	87	179	3906	862	171	5,15	44,90	22,25	United Kingdom	Western Europe
19	Sports Medicine - Open	1,683	Q1	54	132	365	9462	1996	349	5,76	71,68	32,67	United Kingdom	Western Europe
20	Current Reviews in Musculoskeletal Medicine	1,680	Q1	68	61	187	3623	989	187	4,76	59,39	24,58	United States	Northern America
21	Journal of Bone and Joint Surgery	1,666	Q1	315	406	1255	11615	4074	909	2,83	28,61	24,07	United States	Northern America
22	Biology of Sport	1,645	Q1	50	107	312	5058	1715	312	5,53	47,27	19,35	Poland	Eastern Europe
23	Journal of Orthopaedic Translation	1,641	Q1	60	120	294	7809	2234	264	8,38	65,08	31,54	Singapore	Asiatic Region
24	International Journal of Sports Physiology and Performance	1,610	Q1	102	185	698	5881	2659	658	4,15	31,79	21,35	United States	Northern America
25	Bone and Joint Open	1,517	Q1	26	138	380	4403	1265	377	3,25	31,91	23,30	United Kingdom	Western Europe
26	Scandinavian Journal of Medicine and Science in Sports	1,494	Q1	150	280	619	12442	2730	589	4,18	44,44	32,45	Denmark	Western Europe
27	Annals of Physical and Rehabilitation Medicine	1,472	Q1	68	57	305	2221	1151	200	3,68	38,96	47,02	France	Western Europe
28	Journal of ISAKOS	1,472	Q1	18	191	190	7302	671	170	3,06	38,23	23,08	United Kingdom	Western Europe
29	Medicine and Science in Sports and Exercise	1,442	Q1	271	314	788	15469	3143	762	3,90	49,26	36,35	United States	Northern America
30	Arthroplasty	1,378	Q1	17	61	160	2246	621	158	4,44	36,82	18,60	United Kingdom	Western Europe
31	Bone and Joint Research	1,376	Q1	55	67	260	3323	1130	241	4,45	49,60	25,98	United Kingdom	Western Europe
32	Foot and Ankle International	1,375	Q1	133	177	567	4921	1655	510	2,79	27,80	26,55	United States	Northern America
33	Sports Health	1,366	Q1	74	218	296	8804	1199	265	3,80	40,39	32,78	United States	Northern America
34	Orthopaedics and Traumatology: Surgery and Research	1,354	Q1	84	297	838	8736	2553	771	2,79	29,41	19,98	France	Western Europe
35	Journal of Sport Management	1,352	Q1	89	37	135	2393	619	131	4,30	64,68	42,55	United States	Northern America
36	Therapeutic Advances in Musculoskeletal Disease	1,348	Q1	54	57	264	3263	1113	260	4,14	57,25	45,28	United Kingdom	Western Europe
37	Exercise and Sport Sciences Reviews	1,346	Q1	122	29	88	1778	373	75	4,27	61,31	46,99	United States	Northern America
38	Sport, Education and Society	1,340	Q1	83	160	238	8963	1146	230	4,73	56,02	47,58	United Kingdom	Western Europe
39	Clinical Orthopaedics and Related Research	1,339	Q1	244	482	1323	8614	2478	666	1,85	17,87	27,06	United States	Northern America
40	BMJ Open Sport and Exercise Medicine	1,330	Q1	51	186	366	6960	1458	339	3,19	37,42	40,55	United Kingdom	Western Europe

41	Journal of Science and Medicine in Sport	1,319	Q1	133	154	529	5061	1846	474	3,15	32,86	36,53	United Kingdom	Western Europe
42	European Physical Education Review	1,306	Q1	59	65	147	3687	794	147	4,66	56,72	37,83	United Kingdom	Western Europe
43	Journal of Orthopaedics and Traumatology	1,264	Q1	55	71	175	2791	699	175	4,07	39,31	26,34	Italy	Western Europe
44	Journal of the American Academy of Orthopaedic Surgeons	1,260	Q1	145	381	892	11450	2903	848	3,08	30,05	21,89	United States	Northern America
45	Orthopaedic Journal of Sports Medicine	1,258	Q1	68	473	1540	16756	4775	1507	2,57	35,42	22,55	United Kingdom	Western Europe
46	Physical Education and Sport Pedagogy	1,233	Q1	59	92	184	4998	864	184	4,31	54,33	43,43	United Kingdom	Western Europe
47	Calcified Tissue International	1,230	Q1	137	152	393	6876	1560	372	3,67	45,24	43,56	United States	Northern America
48	Spine	1,216	Q1	300	396	1057	11395	3246	958	3,30	28,78	20,09	United States	Northern America
49	Journal of Strength and Conditioning Research	1,188	Q1	174	384	1383	15799	4461	1380	3,12	41,14	21,23	United States	Northern America
50	JOR Spine	1,170	Q1	28	99	140	5438	655	127	3,99	54,93	28,80	United Kingdom	Western Europe
51	European Journal of Sport Science	1,161	Q1	87	178	598	7935	2264	597	3,50	44,58	31,68	United Kingdom	Western Europe
52	Global Spine Journal	1,153	Q1	59	485	842	15319	2718	772	3,31	31,59	19,59	United States	Northern America
53	Acta Orthopaedica	1,150	Q1	136	109	341	2897	888	316	2,22	26,58	29,61	United Kingdom	Western Europe
54	JBMR Plus	1,130	Q1	36	142	351	6263	1186	341	2,35	44,11	45,91	United States	Northern America
55	Evidence and Policy	1,118	Q1	46	33	122	1424	345	120	2,89	43,15	57,52	United Kingdom	Western Europe
56	Asian Spine Journal	1,107	Q1	51	105	345	2934	964	326	3,12	27,94	13,03	South Korea	Asiatic Region
57	Journal of Athletic Training	1,106	Q1	139	149	409	4975	1214	392	2,66	33,39	45,98	United States	Northern America
58	Journal of Physical Activity and Health	1,082	Q1	93	163	432	6843	1179	396	2,17	41,98	52,92	United States	Northern America
59	European Spine Journal	1,076	Q1	173	574	1293	15884	3961	1176	3,00	27,67	25,33	Germany	Western Europe
60	Strength and Conditioning Journal	1,075	Q1	63	83	200	5046	646	199	2,97	60,80	18,00	United States	Northern America
61	Journal of Sports Science and Medicine	1,072	Q1	87	82	242	3953	843	237	3,04	48,21	20,12	Turkey	Middle East
62	European Journal of Applied Physiology	1,060	Q1	167	351	728	20732	2427	682	3,01	59,07	28,18	Germany	Western Europe
63	Archives of Orthopaedic and Trauma Surgery	1,050	Q1	98	558	1471	19302	4051	1462	2,58	34,59	20,03	Germany	Western Europe
64	International Orthopaedics	1,036	Q1	116	388	1141	11394	3030	967	2,60	29,37	20,26	Germany	Western Europe
65	Journal of Sports Sciences	1,020	Q1	171	275	817	13380	2627	803	2,46	48,65	28,68	United Kingdom	Western Europe
66	Physical Therapy in Sport	1,002	Q1	66	93	393	4266	1137	391	2,57	45,87	34,69	United States	Northern America
67	Foot and Ankle Surgery	0,992	Q1	56	133	498	4027	1231	462	2,37	30,28	26,90	United Kingdom	Western Europe
68	JBJS Reviews	0,984	Q1	45	101	306	5890	810	303	2,22	58,32	26,21	United States	Northern America
69	Journal of Hand Surgery	0,982	Q1	142	326	815	8153	1588	714	1,77	25,01	29,55	United States	Northern America
70	Arthroscopy, Sports Medicine, and Rehabilitation	0,973	Q1	21	184	687	6682	1723	686	2,61	36,32	19,63	United States	Northern America
71	Brazilian Journal of Physical Therapy	0,952	Q1	56	68	222	3002	827	198	3,05	44,15	60,61	Brazil	Latin America
72	North American Spine Society Journal	0,943	Q1	13	88	227	3223	582	206	2,55	36,63	19,83	United States	Northern America
73	European Journal for Sport and Society	0,942	Q1	30	26	62	1648	223	57	3,61	63,38	34,29	United Kingdom	Western Europe
74	Journal of the American Academy of Orthopaedic Surgeons Global	0,940	Q1	27	180	451	4802	1102	443	2,05	26,68	24,51	United States	Northern America
75	Journal of Bone and Joint Infection	0,939	Q1	30	33	108	918	272	103	2,74	27,82	38,11	Germany	Western Europe
76	Archives of Osteoporosis	0,938	Q1	49	115	462	4383	1326	450	2,40	38,11	43,53	United Kingdom	Western Europe
77	Journal of Experimental Orthopaedics	0,937	Q1	39	204	381	7726	1064	376	2,74	37,87	20,31	Germany	Western Europe
78	Sports	0,922	Q1	48	357	623	18868	2166	617	3,29	52,85	30,58	Switzerland	Western Europe
79	Arthroplasty Today	0,918	Q1	32	283	678	7907	1320	645	1,94	27,94	19,15	United States	Northern America
80	Journal of Orthopaedic Research	0,907	Q1	179	285	823	11655	2490	810	2,87	40,89	28,72	United States	Northern America
81	Journal of Orthopaedic Trauma	0,903	Q1	150	217	751	5708	1482	695	1,91	26,30	24,21	United States	Northern America
82	Research in Sports Medicine	0,892	Q1	50	88	175	3287	514	175	2,62	37,35	32,42	United Kingdom	Western Europe

83	Journal of Orthopaedic Surgery and Research	0,877	Q1	75	881	2248	33178	7159	2199	2,93	37,66	27,94	United Kingdom	Western Europe
84	BMC Sports Science, Medicine and Rehabilitation	0,875	Q1	39	248	548	12439	1714	546	3,05	50,16	35,07	United Kingdom	Western Europe
85	Frontiers in Sports and Active Living	0,864	Q2	28	582	1336	29229	3773	1230	2,49	50,22	36,27	Switzerland	Western Europe
86	Journal of Bone and Mineral Metabolism	0,860	Q2	88	91	317	3570	987	298	2,10	39,23	28,91	Japan	Asiatic Region
87	Sports Biomechanics	0,856	Q2	56	339	389	12605	1055	384	2,65	37,18	23,01	United Kingdom	Western Europe
88	Journal of Knee Surgery	0,852	Q2	77	102	686	2868	1312	670	1,68	28,12	18,29	Germany	Western Europe
89	Curriculum Studies in Health and Physical Education	0,839	Q2	26	41	67	2181	173	60	2,49	53,20	57,02	United States	Northern America
90	Journal of Foot and Ankle Research	0,837	Q2	58	61	247	2345	668	237	2,39	38,44	46,84	United Kingdom	Western Europe
91	Measurement in Physical Education and Exercise Science	0,835	Q2	50	40	103	2120	273	103	2,58	53,00	32,00	United States	Northern America
92	International Journal of Sports Medicine	0,833	Q2	131	98	446	4397	1185	440	2,05	44,87	31,99	Germany	Western Europe
93	JSES International	0,822	Q2	28	218	544	7261	1238	536	2,10	33,31	16,58	Netherlands	Western Europe
94	Knee	0,821	Q2	98	167	638	5252	1408	598	2,08	31,45	14,73	Netherlands	Western Europe
95	Sports Medicine and Arthroscopy Review	0,813	Q2	62	35	87	1345	198	77	1,41	38,43	24,49	United States	Northern America
96	HIP International	0,811	Q2	50	110	354	3476	731	352	1,85	31,60	16,56	Italy	Western Europe
97	BMC Musculoskeletal Disorders	0,805	Q2	131	1083	3132	41473	8864	3127	2,58	38,29	30,40	United Kingdom	Western Europe
98	Journal of Functional Morphology and Kinesiology	0,804	Q2	32	282	384	14276	1206	372	2,85	50,62	30,49	Switzerland	Western Europe
99	International Journal of Sport Nutrition and Exercise Metabolism	0,801	Q2	94	45	152	1759	384	144	2,37	39,09	31,27	United States	Northern America
100	Journal of Biomechanics	0,800	Q2	233	431	1282	17191	3796	1269	2,65	39,89	31,66	United Kingdom	Western Europe
101	Osteoarthritis and Cartilage Open	0,788	Q2	18	97	217	4549	585	215	2,72	46,90	42,09	United Kingdom	Western Europe
102	Sports Medicine and Health Science	0,784	Q2	17	65	122	3695	384	112	2,59	56,85	41,42	China	Asiatic Region
103	Gait and Posture	0,778	Q2	179	320	997	12239	2851	983	2,69	38,25	37,97	Netherlands	Western Europe
104	Research Quarterly for Exercise and Sport	0,778	Q2	107	154	288	7584	731	275	2,32	49,25	29,41	United Kingdom	Western Europe
105	Spine Deformity	0,778	Q2	41	236	543	7153	1100	519	1,99	30,31	23,10	Netherlands	Western Europe
106	Clinical Spine Surgery	0,774	Q2	45	214	490	6877	977	486	1,90	32,14	18,15	United States	Northern America
107	International Journal of Sports Physical Therapy	0,770	Q2	22	158	452	5952	1018	411	1,92	37,67	32,84	United States	Northern America
108	Sociology of Sport Journal	0,763	Q2	70	41	126	2667	306	121	2,65	65,05	47,19	United States	Northern America
109	Injury	0,762	Q2	151	678	2044	21363	4743	1804	2,01	31,51	29,39	United Kingdom	Western Europe
110	European Journal of Trauma and Emergency Surgery	0,757	Q2	54	343	1054	11916	2555	1013	2,41	34,74	28,37	Germany	Western Europe
111	European Journal of Orthopaedic Surgery and Traumatology	0,752	Q2	52	490	843	13862	1669	824	1,90	28,29	18,60	France	Western Europe
112	Bone Reports	0,750	Q2	35	87	285	4373	687	283	2,51	50,26	43,42	United States	Northern America
113	International Journal of Spine Surgery	0,748	Q2	40	113	477	3317	999	457	1,72	29,35	15,26	United States	Northern America
114	HSS Journal	0,747	Q2	50	109	195	3531	370	161	1,68	32,39	31,67	United States	Northern America
115	CiOS Clinics in Orthopedic Surgery	0,745	Q2	52	122	278	3352	621	274	2,16	27,48	18,16	South Korea	Asiatic Region
116	Disability and Rehabilitation: Assistive Technology	0,745	Q2	59	367	370	18311	1385	365	3,33	49,89	56,16	United Kingdom	Western Europe
117	Hand	0,745	Q2	50	342	613	8784	1075	600	1,63	25,68	27,36	United States	Northern America
118	Physician and Sportsmedicine	0,742	Q2	58	92	217	4070	555	210	2,34	44,24	24,79	United Kingdom	Western Europe
119	SICOT-J	0,737	Q2	30	56	145	1887	301	145	1,79	33,70	12,12	France	Western Europe
120	Archives of Bone and Joint Surgery	0,736	Q2	32	120	359	3511	683	337	1,84	29,26	20,86	Iran	Middle East
121	Journal of Teaching in Physical Education	0,724	Q2	73	77	228	3817	564	224	1,95	49,57	41,70	United States	Northern America
122	Orthopaedic Surgery	0,724	Q2	46	334	1030	11803	2596	1027	2,31	35,34	29,16	Brazil	Latin America
123	Clinical Journal of Sport Medicine	0,720	Q2	121	134	434	4372	899	413	1,82	32,63	34,67	United States	Northern America
124	Foot and Ankle Clinics	0,720	Q2	69	91	189	3116	339	159	1,62	34,24	15,44	United States	Northern America

125	World Journal of Orthopedics	0,717	Q2	64	139	288	4678	742	287	2,47	33,65	23,30	China	Asiatic Region
126	Journal of Shoulder and Elbow Arthroplasty	0,716	Q2	8	18	58	472	124	58	1,98	26,22	25,26	United Kingdom	Western Europe
127	Foot and Ankle Orthopaedics	0,710	Q2	14	119	287	3013	463	270	1,42	25,32	21,92	United States	Northern America
128	Clinics in Shoulder and Elbow	0,699	Q2	6	66	63	2086	119	58	1,89	31,61	15,63	South Korea	Asiatic Region
129	Orthopedic Clinics of North America	0,694	Q2	103	54	170	2537	348	144	2,01	46,98	20,86	United States	Northern America
130	Connective Tissue Research	0,692	Q2	81	40	160	2306	389	156	2,36	57,65	41,32	United Kingdom	Western Europe
131	Orthopedic Reviews	0,690	Q2	28	8	208	320	519	207	2,10	40,00	8,89	United States	Northern America
132	Journal of Spine Surgery	0,683	Q2	30	78	187	2538	337	156	1,49	32,54	17,75	China	Asiatic Region
133	Orthopedic Research and Reviews	0,679	Q2	23	31	104	1241	264	104	2,45	40,03	25,38	New Zealand	Pacific Region
134	Shoulder and Elbow	0,674	Q2	33	160	313	5164	523	273	1,43	32,28	21,57	United Kingdom	Western Europe
135	Journal of Clinical Orthopaedics and Trauma	0,672	Q2	45	174	733	5412	1572	709	1,63	31,10	19,58	Netherlands	Western Europe
136	Translational Journal of the American College of Sports Medicine	0,671	Q2	8	43	75	1619	143	71	1,56	37,65	51,71	United States	Northern America
137	Journal of Orthopaedics	0,670	Q2	41	289	631	8821	1201	610	1,68	30,52	17,95	India	Asiatic Region
138	Musculoskeletal Surgery	0,670	Q2	47	83	146	2548	296	143	1,88	30,70	21,12	Germany	Western Europe
139	Journal of Physiological Anthropology	0,668	Q2	63	33	92	1609	232	90	2,39	48,76	33,33	United Kingdom	Western Europe
140	Journal of Pediatric Orthopaedics	0,655	Q2	114	311	859	7030	1418	795	1,61	22,60	30,83	United States	Northern America
141	German Journal of Exercise and Sport Research	0,654	Q2	21	91	175	4921	379	160	2,16	54,08	41,79	Germany	Western Europe
142	Journal of Foot and Ankle Surgery	0,651	Q2	81	180	691	4553	1088	642	1,48	25,29	23,99	United States	Northern America
143	Foot and Ankle Specialist	0,648	Q2	39	138	241	3718	375	239	1,54	26,94	20,68	United Kingdom	Western Europe
144	Joint Diseases and Related Surgery	0,643	Q2	23	61	345	1483	567	339	1,77	24,31	18,91	Turkey	Middle East
145	Open Access Journal of Sports Medicine	0,643	Q2	12	20	37	1028	75	37	1,65	51,40	19,42	United Kingdom	Western Europe
146	Hip and Pelvis	0,632	Q2	13	36	92	1392	174	91	1,80	38,67	13,16	South Korea	Asiatic Region
147	Journal of Clinical Densitometry	0,630	Q2	85	57	207	1826	364	195	1,58	32,04	45,73	United States	Northern America
148	Arthroscopy Techniques	0,622	Q2	48	472	1047	7464	1516	1047	1,39	15,81	16,70	Netherlands	Western Europe
149	Current Sports Medicine Reports	0,618	Q2	69	83	321	1220	536	285	1,05	14,70	32,00	United States	Northern America
150	Geriatric Orthopaedic Surgery and Rehabilitation	0,614	Q2	40	48	254	1696	449	240	1,48	35,33	31,34	United States	Northern America
151	Human Movement Science	0,614	Q2	109	101	299	4878	685	294	2,01	48,30	39,68	Netherlands	Western Europe
152	Journal of Bone Metabolism	0,611	Q2	18	32	109	1437	236	106	1,96	44,91	42,65	South Korea	Asiatic Region
153	International Journal of Performance Analysis in Sport	0,604	Q2	52	104	177	4190	434	177	2,18	40,29	17,28	United Kingdom	Western Europe
154	Clinics in Sports Medicine	0,600	Q2	96	77	194	4139	351	158	1,50	53,75	29,83	United States	Northern America
155	Journal of Sport Rehabilitation	0,600	Q2	66	91	453	2834	835	451	1,71	31,14	35,21	United States	Northern America
156	Musculoskeletal Care	0,600	Q2	38	147	316	5354	617	315	1,85	36,42	53,68	United States	Northern America
157	Asia-Pacific Journal of Sports Medicine, Arthroscopy, Rehabilitation	0,597	Q2	18	38	71	1276	107	71	1,78	33,58	20,67	Singapore	Asiatic Region
158	Journal of Exercise Rehabilitation	0,595	Q2	32	37	170	934	313	155	1,68	25,24	29,27	South Korea	Asiatic Region
159	Patient Safety in Surgery	0,587	Q2	36	37	105	1335	237	96	2,04	36,08	37,11	United Kingdom	Western Europe
160	JBJS Essential Surgical Techniques	0,585	Q2	22	25	85	302	127	81	1,57	12,08	12,50	United States	Northern America
161	Chinese Journal of Traumatology - English Edition	0,567	Q2	39	88	206	3107	457	200	1,88	35,31	28,39	Netherlands	Western Europe
162	Operative Orthopadie und Traumatologie	0,538	Q2	34	43	139	672	168	120	1,08	15,63	10,48	Germany	Western Europe
163	Journal of Children's Orthopaedics	0,532	Q2	54	50	221	1674	373	204	1,60	33,48	28,67	Germany	Western Europe
164	Journal of Musculoskeletal Neuronal Interactions	0,527	Q2	74	55	181	1676	370	177	1,97	30,47	35,45	Greece	Western Europe
165	Translational Sports Medicine	0,527	Q2	16	12	122	524	161	118	1,48	43,67	30,19	United Kingdom	Western Europe
166	OTA International	0,526	Q2	13	59	198	1648	305	188	1,29	27,93	19,95	United Kingdom	Western Europe

167	Spine Surgery and Related Research	0,523	Q2	21	89	277	2651	410	245	1,52	29,79	11,13	Japan	Asiatic Region
168	Journal of Orthopaedic Science	0,515	Q2	86	338	679	8217	1053	650	1,41	24,31	13,12	Netherlands	Western Europe
169	Pediatric Exercise Science	0,515	Q3	75	39	85	1584	135	84	1,29	40,62	41,38	United States	Northern America
170	Advances in Orthopedics	0,513	Q3	25	16	100	579	173	100	1,76	36,19	26,14	Egypt	Africa/Middle East
171	Montenegrin Journal of Sports Science and Medicine	0,513	Q3	16	20	60	737	125	60	2,15	36,85	20,00	Montenegro	Eastern Europe
172	Current Trauma Reports	0,500	Q3	16	10	57	643	110	57	2,23	64,30	50,00	Switzerland	Western Europe
173	European Journal of Translational Myology	0,496	Q3	19	71	206	2335	417	206	2,22	32,89	39,35	Italy	Western Europe
174	Journal of Back and Musculoskeletal Rehabilitation	0,495	Q3	44	167	428	6097	732	408	1,54	36,51	36,26	Netherlands	Western Europe
175	Journal of Sports Medicine and Physical Fitness	0,495	Q3	79	170	625	6500	904	593	1,42	38,24	29,35	Italy	Western Europe
176	Sport Sciences for Health	0,493	Q3	27	216	400	9236	626	390	1,46	42,76	31,68	Italy	Western Europe
177	Journal of Motor Behavior	0,488	Q3	82	75	181	3693	309	181	1,37	49,24	34,38	United States	Northern America
178	Journal of Hand Surgery Global Online	0,474	Q3	11	200	324	4894	437	314	1,27	24,47	28,57	United States	Northern America
179	Orthopedics	0,473	Q3	81	114	381	3067	457	378	1,20	26,90	20,29	United States	Northern America
180	Clinical Biomechanics	0,471	Q3	152	163	613	6263	1030	608	1,57	38,42	30,39	United Kingdom	Western Europe
181	International Journal of Orthopaedic and Trauma Nursing	0,471	Q3	24	55	105	1813	179	89	1,65	32,96	65,26	United Kingdom	Western Europe
182	Journal of Applied Biomechanics	0,471	Q3	71	61	196	2335	263	190	1,42	38,28	43,37	United States	Northern America
183	JSES Reviews, Reports, and Techniques	0,470	Q3	9	131	264	4008	338	264	1,27	30,60	17,24	United States	Northern America
184	Footwear Science	0,466	Q3	32	31	111	1201	186	106	3,00	38,74	33,03	United Kingdom	Western Europe
185	Hand Surgery and Rehabilitation	0,461	Q3	39	163	422	4229	495	371	1,00	25,94	30,20	France	Western Europe
186	Hand Clinics	0,452	Q3	69	62	187	2487	266	161	1,09	40,11	22,61	United States	Northern America
187	Strategies in Trauma and Limb Reconstruction	0,450	Q3	34	20	93	472	121	89	1,22	23,60	22,84	Italy	Western Europe
188	Journal of Motor Learning and Development	0,446	Q3	22	36	96	1812	126	94	1,44	50,33	50,99	United States	Northern America
189	Journal of Science in Sport and Exercise	0,445	Q3	13	90	136	4253	179	131	1,32	47,26	31,06	United States	Northern America
190	Physical Therapy Reviews	0,443	Q3	40	26	106	1330	154	104	1,45	51,15	48,74	United Kingdom	Western Europe
191	Seminars in Arthroplasty JSES	0,442	Q3	16	129	346	3942	345	345	0,94	30,56	17,27	United States	Northern America
192	Biomechanics (Switzerland)	0,438	Q3	11	60	122	2466	223	120	1,53	41,10	28,87	Switzerland	Western Europe
193	ACSM's Health and Fitness Journal	0,436	Q3	34	80	234	1134	265	196	1,18	14,18	56,20	United States	Northern America
194	Indian Journal of Orthopaedics	0,436	Q3	50	230	784	6381	1006	750	1,18	27,74	17,27	India	Asiatic Region
195	Foot	0,425	Q3	46	58	238	2329	366	236	1,69	40,16	24,27	United Kingdom	Western Europe
196	JSAMS Plus	0,424	Q3	5	25	36	841	58	34	1,61	33,64	51,92	Netherlands	Western Europe
197	Journal of Pediatric Orthopaedics Part B	0,422	Q3	61	117	332	2844	372	310	1,07	24,31	24,15	United States	Northern America
198	Acta Orthopaedica et Traumatologica Turcica	0,408	Q3	41	64	248	1586	281	226	1,04	24,78	17,51	Turkey	Middle East
199	Apunts Sports Medicine	0,391	Q3	18	26	62	995	86	54	1,21	38,27	24,76	Spain	Western Europe
200	Journal of Cartilage and Joint Preservation	0,391	Q3	6	52	110	2378	145	98	1,40	45,73	20,16	Netherlands	Western Europe
201	Physical Activity Review	0,384	Q3	14	25	85	991	130	84	1,55	39,64	35,05	Poland	Eastern Europe
202	Journal of Lasers in Medical Sciences	0,382	Q3	36	69	242	2481	440	241	1,49	35,96	51,44	Iran	Middle East
203	Clinical and Translational Metabolism	0,380	Q3	32	13	11	720	21	11	2,50	55,38	56,41	United States	Northern America
204	Biomedical Human Kinetics	0,361	Q3	12	36	100	1540	121	99	1,13	42,78	33,58	Poland	Eastern Europe
205	Science and Sports	0,358	Q3	32	93	347	2964	381	301	1,05	31,87	29,09	France	Western Europe
206	Deutsche Zeitschrift fur Sportmedizin	0,356	Q3	23	37	112	1346	106	86	1,00	36,38	35,71	Germany	Western Europe
207	Physiology International	0,356	Q3	38	18	89	584	187	89	2,31	32,44	50,88	Hungary	Eastern Europe
208	Physical Activity and Nutrition	0,353	Q3	9	36	89	1471	116	88	1,05	40,86	41,18	South Korea	Asiatic Region

209	Retos	0,338	Q3	33	1291	1300	61341	3565	1300	2,82	47,51	34,82	Spain	Western Europe
210	Pedagogy of Physical Culture and Sports	0,334	Q3	11	58	157	2321	293	157	2,20	40,02	30,14	Ukraine	Eastern Europe
211	Alter	0,331	Q3	27	0	28	0	53	24	0,00	0,00	0,00	France	Western Europe
212	Health, Sport, Rehabilitation	0,331	Q3	8	35	91	1181	147	91	1,52	33,74	45,07	Ukraine	Eastern Europe
213	Physical Activity and Health	0,331	Q3	12	18	73	893	112	73	1,31	49,61	40,98	United Kingdom	Western Europe
214	South African Journal of Sports Medicine	0,330	Q3	8	18	89	335	93	86	1,06	18,61	40,00	South Africa	Africa
215	International Journal of Kinesiology and Sports Science	0,329	Q3	9	27	83	977	106	83	1,33	36,19	34,69	Australia	Pacific Region
216	Techniques in Hand and Upper Extremity Surgery	0,328	Q3	36	28	172	91	105	158	0,61	3,25	17,57	United States	Northern America
217	Annals of Joint	0,324	Q3	10	57	150	2116	148	132	1,09	37,12	16,46	China	Asiatic Region
218	Isokinetics and Exercise Science	0,323	Q3	33	36	119	1586	111	118	0,78	44,06	25,53	Netherlands	Western Europe
219	Current Physical Medicine and Rehabilitation Reports	0,316	Q3	25	44	119	2787	150	119	1,35	63,34	48,70	United States	Northern America
220	Seminars in Musculoskeletal Radiology	0,314	Q3	57	81	222	4313	255	204	0,99	53,25	31,58	United States	Northern America
221	Sports Engineering	0,310	Q3	29	39	100	1196	147	96	1,55	30,67	16,67	United States	Northern America
222	Clinics in Podiatric Medicine and Surgery	0,309	Q3	42	71	188	2172	173	153	0,90	30,59	21,24	United States	Northern America
223	Journal of Hand Surgery Asian-Pacific Volume	0,307	Q3	33	94	385	1716	248	373	0,54	18,26	26,92	Singapore	Asiatic Region
224	Acta Orthopaedica Belgica	0,302	Q3	60	102	330	2823	254	329	0,74	27,68	26,07	Belgium	Western Europe
225	Journal of the American Podiatric Medical Association	0,300	Q3	67	113	369	2831	273	352	0,65	25,05	34,44	United States	Northern America
226	Malaysian Orthopaedic Journal	0,294	Q3	17	50	213	984	149	194	0,66	19,68	21,49	Malaysia	Asiatic Region
227	Journal of Physical Education, Recreation and Dance	0,293	Q3	18	102	278	1571	188	196	0,65	15,40	54,00	United Kingdom	Western Europe
228	Trends in Sport Sciences	0,292	Q3	10	24	68	755	63	68	1,16	31,46	20,00	Poland	Eastern Europe
229	Operative Techniques in Sports Medicine	0,284	Q3	38	39	117	1856	87	105	0,67	47,59	20,54	United States	Northern America
230	Zeitschrift fur Orthopadie und Unfallchirurgie	0,277	Q3	40	74	371	955	207	284	0,53	12,91	25,54	Germany	Western Europe
231	Science of Gymnastics Journal	0,276	Q3	15	42	106	1302	67	93	0,60	31,00	40,71	Slovenia	Eastern Europe
232	Muscles, Ligaments and Tendons Journal	0,275	Q3	53	67	238	2740	191	237	0,75	40,90	39,53	Italy	Western Europe
233	Journal of Sport and Health Research	0,272	Q3	13	41	154	1722	153	154	0,93	42,00	29,80	Spain	Western Europe
234	Hand Therapy	0,271	Q3	17	18	38	888	42	38	0,64	49,33	59,32	United Kingdom	Western Europe
235	Journal of Kinesiology and Exercise Sciences	0,271	Q3	3	28	21	1126	19	21	0,90	40,21	36,27	Poland	Eastern Europe
236	International Journal of Human Movement and Sports Sciences	0,269	Q3	19	110	534	4566	872	534	1,62	41,51	35,31	United States	Northern America
237	Journal of Minimally Invasive Spine Surgery and Technique	0,268	Q3	5	59	120	1361	69	112	0,54	23,07	16,89	South Korea	Asiatic Region
238	Revista Espanola de Cirugia Ortopedica y Traumatologia	0,264	Q3	20	102	259	2590	176	216	0,59	25,39	25,00	Spain	Western Europe
239	Bulletin of the Hospital for Joint Diseases	0,257	Q3	51	44	155	1483	78	138	0,51	33,70	25,50	United States	Northern America
240	Revista Brasileira de Ortopedia	0,257	Q3	29	113	372	3249	228	360	0,47	28,75	21,76	Brazil	Latin America
241	Acta Ortopedica Brasileira	0,253	Q3	30	70	262	1468	172	261	0,54	20,97	24,90	Brazil	Latin America
242	Journal of Prosthetics and Orthotics	0,252	Q3	41	80	162	2206	111	140	0,68	27,58	43,37	United States	Northern America
243	Orthoplastic Surgery	0,251	Q3	6	16	85	392	57	78	0,71	24,50	22,50	United Kingdom	Western Europe
244	Sport Mont	0,251	Q3	16	60	232	2253	187	232	0,82	37,55	32,69	Montenegro	Eastern Europe
245	Seminars in Spine Surgery	0,249	Q3	19	41	113	2032	60	104	0,55	49,56	17,29	United States	Northern America
246	Strategies	0,245	Q3	15	51	144	663	75	124	0,50	13,00	45,83	United Kingdom	Western Europe
247	Unfallchirurgie (Germany)	0,240	Q3	56	126	527	2802	263	402	0,48	22,24	23,80	Germany	Western Europe
248	International Journal of Athletic Therapy and Training	0,236	Q3	20	55	163	1292	91	158	0,36	23,49	45,90	United States	Northern America
249	Operative Techniques in Orthopaedics	0,235	Q3	33	31	99	1246	38	87	0,29	40,19	32,10	United States	Northern America
250	International Biomechanics	0,231	Q3	14	3	19	79	17	19	0,50	26,33	25,00	United Kingdom	Western Europe

251	Fisioterapia em Movimento	0,229	Q4	6	40	139	1327	64	136	0,42	33,18	60,26	Brazil	Latin America
252	Human Sport Medicine	0,229	Q3	10	121	419	2148	114	418	0,28	17,75	59,89	Russian Federation	Eastern Europe
253	Acta Chirurgiae Orthopaedicae et Traumatologiae Cechoslovaca	0,226	Q4	31	48	189	1315	107	184	0,43	27,40	20,00	Czech Republic	Eastern Europe
254	Polish Journal of Sport and Tourism	0,226	Q4	9	26	74	974	64	74	0,84	37,46	39,06	Germany	Western Europe
255	Annals of Applied Sport Science	0,225	Q4	12	56	154	2059	155	153	1,03	36,77	31,14	Iran	Middle East
256	Orthopaedic Nursing	0,225	Q4	44	58	182	1118	101	160	0,54	19,28	65,22	United States	Northern America
257	Balneo and PRM Research Journal	0,222	Q4	9	119	225	5158	237	225	1,06	43,34	55,37	Romania	Eastern Europe
258	Journal of Orthopaedics, Trauma and Rehabilitation	0,221	Q4	9	51	116	1270	58	116	0,56	24,90	28,49	Netherlands	Western Europe
259	Orthopadie	0,215	Q4	48	121	443	3508	225	397	0,52	28,99	22,89	Germany	Western Europe
260	Medicina dello Sport	0,212	Q4	17	42	168	1532	83	160	0,63	36,48	41,26	Italy	Western Europe
261	Movement and Sports Sciences - Science et Motricite	0,212	Q4	17	32	83	1931	42	78	0,44	60,34	29,00	France	Western Europe
262	Handchirurgie Mikrochirurgie Plastische Chirurgie	0,211	Q4	34	58	256	1061	110	228	0,41	18,29	23,64	Germany	Western Europe
263	Motricidade	0,211	Q4	17	38	175	1134	84	168	0,41	29,84	44,76	Portugal	Western Europe
264	Assistive Technology Outcomes and Benefits	0,210	Q4	5	11	34	301	31	30	0,69	27,36	68,42	United States	Northern America
265	Open Sports Sciences Journal	0,209	Q4	18	9	57	475	42	55	0,65	52,78	48,15	United Arab Emirates	Middle East
266	Physical Treatments	0,209	Q4	5	32	84	1162	56	83	0,61	36,31	39,78	Iran	Middle East
267	Journal of Arthroscopy and Joint Surgery	0,202	Q4	10	37	126	906	44	121	0,22	24,49	8,47	India	Asiatic Region
268	Series on Biomechanics	0,202	Q4	8	43	129	1078	67	129	0,59	25,07	46,09	Bulgaria	Eastern Europe
269	Orthopaedics, Traumatology and Prosthetics	0,201	Q4	3	53	96	1307	24	92	0,25	24,66	17,16	Ukraine	Eastern Europe
270	Comparative Exercise Physiology	0,198	Q4	20	39	160	1433	88	158	0,47	36,74	45,28	Netherlands	Western Europe
271	Ortopedia Traumatologia Rehabilitacja	0,196	Q4	27	15	113	0	63	113	0,49	0,00	22,00	Poland	Eastern Europe
272	Chinese Journal of Microsurgery	0,193	Q4	6	67	390	1475	135	382	0,39	22,01	30,81	China	Asiatic Region
273	Turkish Journal of Physiotherapy and Rehabilitation	0,193	Q4	6	42	117	1445	50	117	0,30	34,40	59,60	Turkey	Middle East
274	Asian Journal of Sports Medicine	0,191	Q4	39	29	92	1238	59	85	0,69	42,69	41,25	Iran	Middle East
275	Central European Journal of Sport Sciences and Medicine	0,190	Q4	5	23	99	811	49	99	0,36	35,26	26,83	Poland	Eastern Europe
276	MHSalud	0,189	Q4	6	20	63	853	42	63	0,68	42,65	17,74	Costa Rica	Latin America
277	Orthopaedics and Trauma	0,184	Q4	32	71	186	1468	87	136	0,48	20,68	38,46	United Kingdom	Western Europe
278	Obere Extremitat	0,182	Q4	16	57	135	1142	37	107	0,22	20,04	14,81	Germany	Western Europe
279	Current Orthopaedic Practice	0,179	Q4	27	49	293	1388	84	284	0,27	28,33	23,83	United States	Northern America
280	Sportverletzung-Sportschaden	0,179	Q4	25	72	243	437	32	82	0,08	6,07	12,96	Germany	Western Europe
281	Sport TK	0,178	Q4	10	120	263	3654	182	263	0,69	30,45	31,40	Spain	Western Europe
282	Trauma Case Reports	0,178	Q4	13	128	531	1721	224	530	0,36	13,45	22,38	United Kingdom	Western Europe
283	Reabilitacijos Mokslai: Slauga, Kineziterapija, Ergoterapija	0,176	Q4	2	20	60	452	46	60	1,15	22,60	75,00	Lithuania	Eastern Europe
284	Studia Sportiva	0,176	Q4	4	34	86	1299	45	86	0,53	38,21	34,69	Czech Republic	Eastern Europe
285	JBJS Case Connector	0,175	Q4	15	273	950	4215	300	948	0,31	15,44	16,02	United States	Northern America
286	Orthopedic Journal of China	0,167	Q4	5	412	941	9751	566	941	0,60	23,67	24,84	China	Asiatic Region
287	Pamukkale Journal of Sport Sciences	0,166	Q4	3	32	42	1662	11	42	0,32	51,94	39,78	Turkey	Middle East
288	Journal of Hard Tissue Biology	0,163	Q4	18	30	122	878	48	114	0,38	29,27	31,69	Japan	Asiatic Region
289	Techniques in Orthopaedics	0,163	Q4	27	48	222	705	52	217	0,18	14,69	16,27	United States	Northern America
290	Physiotherapy Practice and Research	0,160	Q4	12	18	80	588	33	75	0,35	32,67	56,14	Netherlands	Western Europe
291	Sports Orthopaedics and Traumatology	0,160	Q4	15	48	140	862	41	102	0,30	17,96	26,32	Germany	Western Europe
292	Clinical Osteology	0,159	Q4	3	17	63	336	7	53	0,05	19,76	37,50	Czech Republic	Eastern Europe

293	International Journal of Disabilities Sports and Health Sciences	0,158	Q4	6	125	137	4482	73	137	0,53	35,86	32,34	Turkey	Middle East
294	Chinese Journal of Orthopaedic Trauma	0,157	Q4	9	131	574	3525	208	561	0,36	26,91	27,08	China	Asiatic Region
295	Indian Spine Journal	0,155	Q4	4	34	103	919	14	97	0,13	27,03	8,74	India	Asiatic Region
296	Archivos de Medicina del Deporte	0,154	Q4	16	18	134	541	41	118	0,26	30,06	31,48	Spain	Western Europe
297	Journal of Musculoskeletal Surgery and Research	0,154	Q4	7	73	162	1620	53	134	0,27	22,19	30,93	United States	Northern America
298	Sarkomy Kostej, Magkih Tkanej i Opuholi Kozi	0,151	Q4	3	34	69	1043	15	64	0,24	30,68	45,67	Russian Federation	Eastern Europe
299	Travma	0,151	Q4	3	22	126	505	23	126	0,18	22,95	22,00	Ukraine	Eastern Europe
300	Hirurgia Pozvonochnika	0,149	Q4	8	43	127	1169	54	119	0,37	27,19	25,40	Russian Federation	Eastern Europe
301	Chinese Journal of Orthopaedics	0,148	Q4	9	195	600	7105	244	600	0,42	36,44	26,47	China	Asiatic Region
302	Pediatric Traumatology, Orthopaedics and Reconstructive Surgery	0,148	Q4	8	44	144	1693	42	141	0,33	38,48	37,62	Russian Federation	Eastern Europe
303	Travmatologiya i Ortopediya Rossii	0,147	Q4	6	66	188	2100	74	174	0,38	31,82	23,11	Russian Federation	Eastern Europe
304	Minerva Orthopedics	0,145	Q4	7	54	211	1985	88	187	0,43	36,76	28,02	Italy	Western Europe
305	Extreme Medicine	0,144	Q4	4	35	160	1024	37	160	0,27	29,26	44,06	Russian Federation	Eastern Europe
306	South African Orthopaedic Journal	0,144	Q4	5	28	107	694	30	89	0,30	24,79	26,53	South Africa	Africa
307	Sports Medicine: Research and Practice	0,141	Q4	4	13	123	516	14	123	0,11	39,69	46,30	Russian Federation	Eastern Europe
308	Techniques in Foot and Ankle Surgery	0,141	Q4	21	38	108	823	13	104	0,14	21,66	20,17	United States	Northern America
309	Fuss und Sprunggelenk	0,139	Q4	11	25	86	984	14	75	0,17	39,36	27,27	Germany	Western Europe
310	Journal de Traumatologie du Sport	0,139	Q4	10	84	116	1613	32	103	0,28	19,20	26,40	France	Western Europe
311	Korean Journal of Sport Science	0,138	Q4	5	66	173	3137	33	173	0,20	47,53	32,61	South Korea	Asiatic Region
312	Genij Ortopedii	0,137	Q4	10	93	317	3371	114	314	0,36	36,25	23,38	Russian Federation	Eastern Europe
313	Sport Science	0,133	Q4	25	0	109	0	78	105	0,15	0,00	0,00	Bosnia and Herzegovina	Eastern Europe
314	Journal of Sport Biomechanics	0,132	Q4	2	24	66	822	13	65	0,17	34,25	42,11	Iran	Middle East
315	Revista Andaluza de Medicina del Deporte	0,132	Q4	20	13	90	428	19	89	0,27	32,92	27,59	Spain	Western Europe
316	Voprosy Kurortologii Fizioterapii i Lechebnoi Fizicheskoi Kultury / P	0,132	Q4	11	70	205	1992	64	205	0,35	28,46	64,49	Russian Federation	Eastern Europe
317	Revista Cubana de Ortopedia y Traumatologia	0,129	Q4	5	49	148	1095	11	137	0,07	22,35	28,74	Cuba	Latin America
318	Chinese Journal of Tissue Engineering Research	0,126	Q4	12	873	2542	33135	525	2539	0,22	37,96	31,22	China	Asiatic Region
319	Chinese Journal of Trauma	0,124	Q4	5	92	473	3942	103	473	0,21	42,85	25,24	China	Asiatic Region
320	Journal of Musculoskeletal Research	0,123	Q4	19	46	113	1118	21	111	0,15	24,30	36,14	Singapore	Asiatic Region
321	Arthroskopie	0,122	Q4	9	72	200	1826	30	167	0,14	25,36	17,39	Germany	Western Europe
322	Sport and Exercise Medicine Switzerland Journal	0,119	Q4	15	39	101	421	7	82	0,07	10,79	28,21	Switzerland	Western Europe
323	Coluna/ Columna	0,118	Q4	10	55	175	1207	24	174	0,10	21,95	17,95	Brazil	Latin America
324	Japanese Journal of Physical Fitness and Sports Medicine	0,118	Q4	14	58	89	439	11	87	0,08	7,57	22,03	Japan	Asiatic Region
325	N.N. Priorov Journal of Traumatology and Orthopedics	0,118	Q4	3	55	110	1900	22	110	0,11	34,55	24,39	Russian Federation	Eastern Europe
326	Journal of Turkish Spinal Surgery	0,117	Q4	4	32	102	757	7	91	0,08	23,66	11,11	Turkey	Middle East
327	Actualizaciones en Osteologia	0,116	Q4	8	6	40	234	2	29	0,07	39,00	62,07	Argentina	Latin America
328	Minerva Ortopedica e Traumatologica	0,115	Q4	9	0	9	0	1	6	0,00	0,00	0,00	Italy	Western Europe
329	Research in Sport Education and Sciences	0,115	Q4	2	21	90	854	8	90	0,15	40,67	30,56	Turkey	Middle East
330	Revue de Chirurgie Orthopedique et Traumatologique	0,115	Q4	49	154	852	4953	53	785	0,06	32,16	22,44	France	Western Europe
331	European Journal of Musculoskeletal Diseases	0,114	Q4	5	75	108	2965	96	86	0,87	39,53	35,06	Italy	Western Europe
332	Journal of Korean Society of Spine Surgery	0,114	Q4	2	16	63	327	3	63	0,07	20,44	7,27	South Korea	Asiatic Region
333	Croatian Sports Medicine Journal	0,107	Q4	1	18	31	545	3	31	0,12	30,28	39,13	Croatia	Eastern Europe
334	Chinese Journal of Rheumatology	0,106	Q4	3	71	209	2170	12	209	0,05	30,56	55,13	China	Asiatic Region

335	Bulletin of the Physical Fitness Research Institute	0,104	Q4	4	0	15	0	0	9	0,00	0,00	0,00	Japan	Asiatic Region
